# Phylloides tumor of the breast: a rare neoplasm, though not that innocent

**DOI:** 10.1186/1477-7800-6-6

**Published:** 2009-02-20

**Authors:** Michael Stamatakos, Sofia Tsaknaki, Konstantinos Kontzoglou, John Gogas, Alkiviades Kostakis, Michael Safioleas

**Affiliations:** 1 Department of Propedeutic Surgery, Medical School, University of Athens, Laiko General Hospital, Athens, Greece; 2 Department of Surgery, Medical School, University of Athens, ATTIKON General Hospital, Athens, Greece

## Abstract

**Background:**

Cystosarcoma phylloides (CP) is an extremely rare form of breast cancer with an unpredictable clinical course. The histological characteristics of this neoplasm have not proved to offer much in the estimation of prognosis of these patients.

**Patients and methods:**

In our clinics, in a time period of 38 years, 22 patients with cystosarcoma phylloides were treated. There were 5 cases of malignancy, 15 cases with benign tumors, and two cases histologically characterized as borderline neoplasia. Metastases were manifested in one patient. All patients were on a 5-year follow-up, except in five cases, one operated three years ago and four operated within the last two years.

**Results:**

16 of 22 patients did not present any signs of local recurrence or metastases. There were three patients that manifested local recurrence and underwent supplementary ongectomy or mastectomy and are free of recurrence ever since. One patient with metastatic CP died.

**Conclusion:**

Independently of its histopathological behavior, CP is a tumor difficult to be treated. Meticulous follow-up is mandatory in order to manage possible recurrence of the neoplasm.

## Background

Cysteosarcoma phylloides (CP) constitutes a rare form of breast neoplasia, which represents less than 1% of total breast neoplasias and only 2,3% of all fibro-epithelial breast tumors [1-4]. It was first described by Muller in 1838, who also introduced the term "cystosarcoma phylloides" because of the extensions of the tumor mimicking the shape of leaves, intruding within the cystic cavities of the tumor [5]. This neoplasm is manifested in women of all ages, including adolescent and the elderly, with the majority ranging between 35 and 54 years [6-10]. Multiple studies have been conducted during the recent years concerning the comprehension of the biological behavior of CP [11,12]. The histological classification of these neoplasms into benign, malignant and borderline has not proven to be successful, because the benign form of cystosarcoma phylloides could manifest metastases, while cases with malignant type of CP finally presented an excellent prognosis [13-15].

## Presentation of our experience

In the time period between 1968 and 2007, 22 women of a mean age of 38 years, presented in our clinics with the diagnosis of cystosarcoma phylloides. Among them 19 were operated for breast tumor, which was proved by the histological examination to be a cystosarcoma phylloides. The remaining three were previously treated in other institutions and were referred to our hospital for further treatment.

The main complaint among these women was a palpable, painless mass of the breast. Two patients presented pain of the breast region, while one patient mentioned tenderness of the respective axilla. In 11 patients the mass was located on the right breast, while in the remaining 11 patients it was found in the left breast. The clinical examination revealed a solitary mobile tumoral lesion in all the patients, measuring between 3–12 cm. Three patients presented with dilated veins of the overlying skin and in one case bluish discoloration of the skin of the breast above the tumor was present. A palpable mass of the respective auxiliary region was observed in two women.

The initial management was surgical in 21 of 22 cases, including tumor resection within healthy borders in 12 patients and simple mastectomy in 5 cases. The two patients with enlarged lymph glands were treated by modified radical mastectomy, and there were two cases where subcutaneous mastectomy and silicone prostheses insertion was performed. Among them, there was a woman who presented at our clinic with the diagnosis of benign cysteosarcoma phylloides, from a recent breast biopsy performed elsewhere, who underwent wide local excision of the remaining tumor. This patient had two local excisions of a benign cytosarcoma phylloides eight and four years previously. There was also one patient who manifested local recurrence of benign CP, having undergone simple mastectomy six months before at another institution. The remaining patient had undergone a previous simple mastectomy three years ago for the management of malignant CP and did not present any signs of local recurrence, but only clinical signs of pulmonary and pleural metastasis.

## Results

Histological examination of the resected breast segment revealed a benign lesion in 15 cases, a malignant tumor in 4 cases, while in the remaining two cases the neoplasm was characterized as borderline. The enlarged lymph nodes resected in two patients were established by the pathologoanatomic examination as presenting signs of reactive hyperplasia, and no metastatic element was identified in them.

The woman who presented with pulmonary and pleural metastasis, established by clinical examination and laboratory control (chest X-rays, pleural aspiration and cytological examination) died three months after the diagnosis.

All the remaining patients were followed-up for 5 years for signs of recurrence. One of the patients with a CP characterized as borderline manifested local recurrence two years after initial ongectomy. She was managed by wide total excision of the tumor, though she came back two years after the second operation with signs of a new recurrence. She refused to undergo a third operation for esthetic reasons and was lost on follow-up. Another woman appeared four years after primary surgical treatment with a locally recurred lesion; a wide local excision was performed. However, the histological examination demonstrated that the tumor was a fibroadenoma, thus this case was not considered as a recurrent CP. The remaining women did not manifest any signs of recurrence during follow-up. The last five patients who were treated in our institution for cystosarcoma fylloides were operated one of them three years ago, two of them one year ago and the remaining two were treated within the year 2007, so they have not yet completed the 5-year follow-up; until today all five are free of recurrence symptoms and signs.

**Figure 1:**
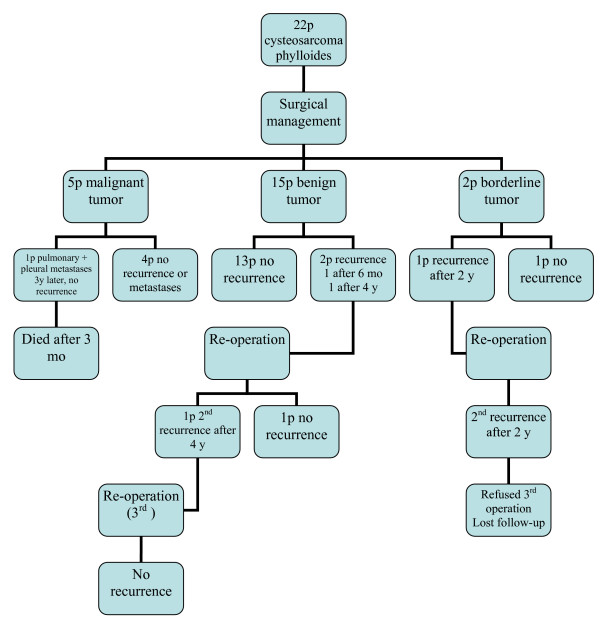
**Clinical course of 22 patients with cysteosarcoma phylloides**.

Figure 1 demonstrates the clinical course of all 22 patients treated in our hospital, since the disease first appeared.

## Discussion

Cystosarcoma phylloides is a rare breast tumor, which presents a rapid development among young women. It usually takes over a quarter or half of the affected breast. On palpation it is mobile with clear borders. Due to the big size of the tumor, the overlying skin is tense, and presents a visible and dilated venous plexus. As a result, skin ulceration may easily be created. Enlargement of the auxiliary lymph nodes is a rare symptom. These tumors are most commonly benign and only 10% of them present a malignant behavior. They do not manifest breast fascia infiltration, though they invade in adjacent tissues. At a rate of 50% these neoplasms give metastasis to the bones and viscera, mainly heamatogenously [16]. Another common characteristic seems to be their frequently observed local recurrences, independently of malignancy [17].

Laboratory control, such as ultrasonography, mammography, etc., in combination with the clinical examination and the self-examination will definitely lead to the recognition of a lesion variably sized. Small tumors may be characterized as fibroadenomas [18], while larger ones look like sarcomas of undefined appearance, create tension on the skin and may present regions of superficial hemorrhage or necrosis. The prognostic value of immunohistochemical indexes for the cystosarcoma phylloides provides very little to a definitive prognosis. This is due to the small number of studies upon this subject, as well as the variety of histological classifications for CP [19-21].

Through the years, all types of breast surgery have been used for the management of cystosarcoma phylloides.

Radical mastectomy is no longer applied, and because the tumor is heamatogenously disseminated, resection of auxiliary lymph nodes is not justified [20,22]. Nowadays, the preferred procedure is local resection of the tumor within healthy borders (1–2 cm) in order to prevent local recurrence [12,23]. A total mastectomy is performed if necessary, such as in cases of large tumors, which occupy the whole breast [23-25]. For cystosarcomas which have already manifested metastases, there is no sufficient treatment available for a successful control of the disease [26]. The role of chemotherapy and radiotherapy remains uncertain [27-30]. Today, however, the studies upon estrogen and progesterone receptors in these tumors seem to provide hopeful results, useful for the hormonal management of CP [30,31].

## Conclusion

Cystosarcoma phylloides is a rare neoplastic condition of the breast, which belongs to the non-epithelial tumors. The classification by authors into benign and malignant is not particularly successful, because the natural history of the tumor is not always related to its histological image. Its equivocal biological behavior may affect the patients' prognosis and survival, and thus it remains an unsolved puzzle, which evolving studies will try to clarify in the future.

## Abbreviations

CP: Cystosarcoma phylloides.

## Consent

Written informed consent was obtained from the patients.

## Competing interests

The authors declare that they have no competing interests.

## Authors' contributions

MS was the surgeon who performed the operations and edited part of the manuscript. ST carried out literature search, revision of bibliography and helped to prepare the draft. KK was the surgeon who performed the operation and edited most of the manuscript. JG was the surgeon who performed the operations. MS was the surgeon who performed the operations.
